# The Effects of an Advanced Air Purification Technology on Environmental and Clinical Outcomes in a Long-Term Care Facility

**DOI:** 10.1093/gerona/glad113

**Published:** 2023-05-03

**Authors:** Alicia R Urrutia, Susan D Schlener, Sherrine Eid, Kelly A Bock, Kathryn C Worrilow

**Affiliations:** LifeAire Systems, Allentown, Pennsylvania, USA; Phoebe Ministries Allentown, Allentown, Pennsylvania, USA; Sherrine Eid Consulting, Macungie, Pennsylvania, USA; Phoebe Ministries Allentown, Allentown, Pennsylvania, USA; LifeAire Systems, Allentown, Pennsylvania, USA

**Keywords:** HAI, HVAC, Infection, Pathogen, VOC

## Abstract

**Background:**

Long-term care facilities (LTCFs) are constantly working to reduce sources of infectious pathogens to improve resident care. LTCF residents are particularly susceptible to health care-associated infections (HAIs), many of which originate from the air. An advanced air purification technology (AAPT) was designed to comprehensively remediate volatile organic compounds (VOCs) and all airborne pathogens including all airborne bacteria, fungi, and viruses. The AAPT contains a unique combination of proprietary filter media, high-dose ultraviolet germicidal irradiation, and high-efficiency particulate air (HEPA) filtration.

**Methods:**

The AAPT was installed in an LTCF’s heating, ventilation, and air-conditioning ductwork and 2 floors were studied: the study floor with comprehensive AAPT remediation and HEPA filtration and the control floor with only HEPA filtration. VOC loading and airborne and surface pathogen loading were measured in 5 locations on both floors. Clinical metrics such as HAI rates were also studied.

**Results:**

There was a statistically significant 98.83% reduction in airborne pathogens, which are responsible for illness and infection, an 89.88% reduction in VOCs, and a 39.6% reduction in HAIs. Surface pathogen loading was reduced in all locations except 1 resident room where the detected pathogens were linked to direct touch.

**Conclusions:**

The removal of airborne and surface pathogens by the AAPT led to a dramatic reduction in HAIs. The comprehensive removal of airborne contaminants has a direct positive impact on resident wellness and quality of life. It is critical that LTCFs incorporate aggressive airborne purification methods with their current infection control protocols.

As of 2022, there are 55.6 million people in the United States aged 65 and older, which accounts for 16.9% of the population (1). This population has been growing over the last 10 years and is expected to reach 94.7 million by 2060 (2,3). In addition to the 65 and older population, the 85 and older population is continuing to grow and is projected to reach 14.4 million in 2040 (3). The need for long-term care facilities (LTCFs) will continue to increase as the population of older adults continues to grow. An LTCF is characterized as a residential or outpatient facility, including skilled nursing facilities, assisted living facilities, nursing homes, or short-term rehabilitation facilities, that provides care to individuals who are unable to care for themselves independently (4,5). An estimated 2.1 million people in the United States live in LTCFs with over 1.2 million living in nursing homes (2,4,6).

Poor indoor air quality has been linked to increased health care-associated infections (HAIs) (7). HAIs are of great concern to LTCF administrators as their residents are at a similar risk for HAIs as acute care facilities (4,8). The risk is similar because of both age-related factors and facility-related factors (9). Individuals residing in LTCFs are particularly susceptible to HAIs due to age-related factors such as alterations in the immune system and organ systems, chronic diseases, functional impairment, malnutrition, invasive devices, and medication use that can individually and collectively increase the likelihood of infection (9,10). Additionally, older adults in nursing homes and other LTCFs are susceptible to facility-related factors that increase their risk of HAIs because they live and socialize in these facilities, and regularly interact in close proximity with other residents, staff, visitors, and health care providers (9–11). It is estimated that there are 1.13–2.68 million infections annually in nursing home residents alone within the United States, with urinary tract infections and pneumonia being the most prevalent (12).

A literature review of infection prevention and control programs in LTCFs identified commonly implemented infection control initiatives used in LTCF settings beyond the standard use of high-efficiency particulate air (HEPA) filtration. Of the different methods identified, hand hygiene as part of the intervention was the most prevalent with other strategies comprising interventions focused on multidrug-resistant organisms, oral care protocols and measures to prevent respiratory tract infections, enhanced surface cleaning, personal protective equipment, additional training and education, and personal protective equipment (13). These infection control programs have been very successful in reducing HAI rates. The World Health Organization reports that such programs are responsible for an excess of 30% reductions in HAIs (14). These protocols do not traditionally go beyond the use of HEPA filters to address the presence of airborne pathogens even though 69%–80% of all pathogens that cause infections are airborne (15).

Respiratory diseases such as chronic obstructive pulmonary disease (COPD), emphysema, or chronic bronchitis affect 10% of the U.S. population aged 65 and older and these conditions are among the leading chronic conditions affecting this population (2). A retrospective database analysis of 126 121 LTCF residents in the United States determined that the prevalence of COPD within the LTCF population is 21.5% (16). Diseases such as COPD have been documented to cause a decline in lung function and increased mortality (17,18). Volatile organic compounds (VOCs) are a broad category of carbon-based chemicals that readily evaporate at room temperature and are a known constituent of air pollution (19). VOCs cause oxidative stress, leading to impairment in pulmonary function, which is particularly dangerous for older adults with already compromised respiratory systems (20,21).

An advanced air purification technology (AAPT) was designed to comprehensively remediate all airborne contaminants before they enter a critical space (22). The AAPT is a 5-stage air purification system that is placed in line with the facility’s heating, ventilation, and air-conditioning (HVAC) ductwork downstream of the air handler. Figure 1 provides a schematic depicting the HVAC layout for both the study floor and the control floor. The AAPT employs 2 stages of proprietary molecular media to remediate all VOCs from the air. After the VOC remediation media is a high-dose ultraviolet germicidal irradiation (UVGI) chamber that takes into account reflectivity, air velocity, air volume, dwell time, and other key factors to provide single-pass remediation of all airborne pathogens. Following the UVGI section is a polishing VOC filter to remove any remaining VOCs. The last stage of the AAPT is an HEPA filter. Figure 1 also shows the 5 stages of the AAPT. Though UVGI, VOC filtration, and HEPA filtration are well-established technologies, the novelty of the AAPT lies in the proprietary nature of the VOC filters and with the unique combination of these components to deliver ultrapure contaminant-free air, not achievable through any one of these technologies alone. Additionally, typical UVGI installations rely on multiple passes to adequately destroy airborne pathogens, while the AAPT delivers a dose sufficient to destroy all airborne pathogens in a single pass through the system. It was important in the design of the system for LTCFs to include both VOC remediation as well as pathogen remediation because the older adult population in these facilities is susceptible to both. Additionally, the AAPT does not produce any intermediate molecules, byproducts, or ozone that could be potentially harmful.

**Figure 1.**
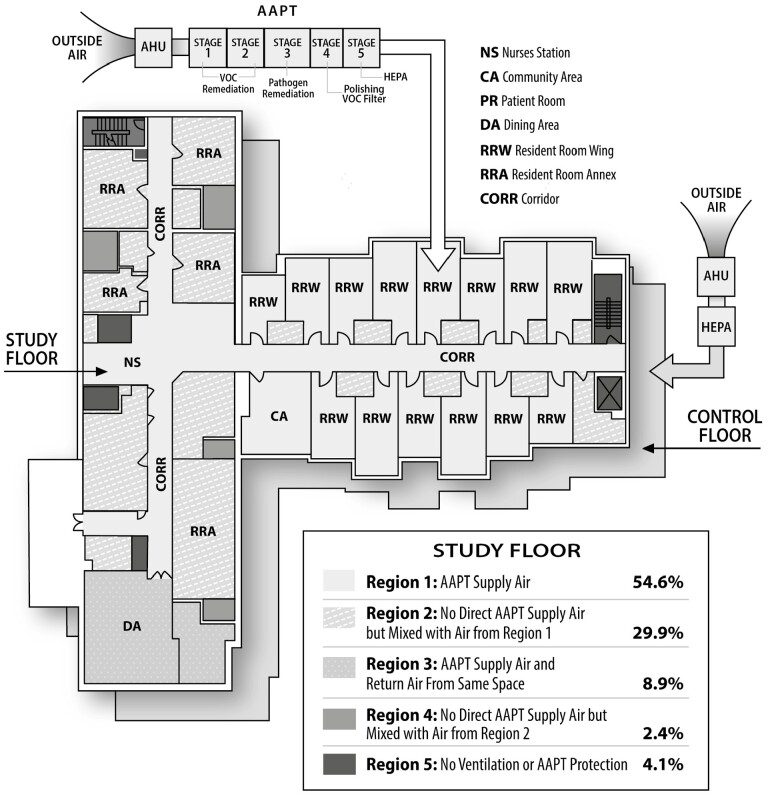
Heating, ventilation, and air-conditioning layout, advanced air purification technology schematic, and study floor air purity. AAPT = advanced air purification technology; AHU = air handling unit; HEPA = high-efficiency particulate air; VOC = volatile organic compounds.

The goal of this study was to understand the effect of the AAPT on key environmental and clinical outcomes compared to HEPA filtration only. It was hypothesized that comprehensive remediation of all airborne contaminants, biological, VOC, and particulate, would reduce the airborne and surface pathogen load, and reduce the airborne VOC load. This hypothesis is supported by the different components of the AAPT. The proprietary VOC media is designed to remove all VOCs from the air entering the protected space and the UVGI section is mathematically and genomically modeled to provide a single pass kill of all harmful airborne pathogens on a single pass. The use of these 2 components in conjunction with HEPA filtration provides complete removal of harmful airborne contaminants. By reducing 3 common vectors of illness, VOCs, pathogens, and particulates, in the residential and clinical environment, it was hypothesized that the rate of illness and infection would be reduced.

## Method

The Institutional Review Board at the LTCF reviewed and approved the study in December 2016. The all-weather rooftop AAPT was installed, through a retrofit project, onto the roof of an existing LTCF. The original HVAC layout was utilized, including all terminal air conditioners and unit ventilators. This layout was representative of a typical LTCF HVAC layout. The research team evaluated 2 air filtration zones on 2 different resident floors between October 2019 and January 2021 (23). The first zone was a control floor with only previously existing HEPA filtration and the second zone was the study floor with HEPA filtration, followed by the AAPT remediation. The HEPA filters used on both the study floor and the control floor were new. Figure 1, described previously, illustrates the layout of the study floor and the associated air purity of each region of the floor. Due to limitations in the HVAC layout within typical LTCF buildings and the requirement to provide individual temperature control for all resident rooms, it was not possible to deliver pure AAPT air to all locations within the study floor. The HVAC system was carefully designed to supply AAPT air with VOC and pathogen remediation to the majority of the floor. Portions of the floor that did not receive AAPT air were designed to be negatively pressured, meaning they pulled air from adjacent spaces, and adjacent to areas with comprehensive AAPT remediated air. This configuration allowed the comprehensive AAPT remediated air to be pulled into the adjacent areas and mixed with the non-AAPT remediated air.

The physical layout for the study floor and control floor were identical and the control floor was located directly below the study floor. The 2 floors were built and renovated at the same time with the same construction materials and contractors. Both the study floor and control floor housed memory support residents. Both floors had similar staffing, standard operating procedures (SOPs), existing HEPA filtration, and cleaning protocols. Care was taken to ensure that the floor staff and residents were blinded to the study. In addition, no new major resident care initiatives or cleaning protocols were introduced on either floor during the study period.

On both the study and control floor, 5 locations were selected for air quality testing and the environmental data were studied prospectively. The sampling sites were chosen based on their locations and the typical activities occurring in these spaces. It was important to the research team to select locations actively frequented by residents and staff and to select a wide range of locations with different functions including resident rooms, the dining areas (DA), and community areas (CA). Two resident rooms were sampled per floor, one labeled the resident room annex (RRA) and the other labeled the resident room wing (RRW). The dimensions of the sampling locations are reported in Table 1. All samples were taken during live operation in occupied rooms. Live operation was determined by the presence of people, residents, and staff, in the rooms during sampling. Sampling on the control floor and study floor occurred concurrently and the locations were sampled on both floors in the same order, at the same time, and for the same duration. In addition, the sampling order was determined based on events throughout the day, for example, the DAs were sampled during lunch time and the CAs were sampled during well-attended activities. Both floors had the same number of residents and staff and the sampling locations were equally populated during sampling. It was important to sample during live operation and in occupied rooms because the amount of airborne contaminants present is affected by the activities and number of individuals in the space. Studies have noted that there can be up to 4 times more bacteria in occupied rooms as compared to unoccupied rooms (7). There were 5 different locations selected for environmental testing on both floors; the corridor (CORR), RRW, RRA, CA, and the DA. VOC canister testing was performed in each of the 5 locations along with airborne fungal and bacterial sampling. Additional airborne fungal and bacterial sampling was performed in the bathroom of both resident rooms. The specific surface samples that were taken in each of the 5 locations included the hallway railing and nurse’s station in the CORR, the TV remote, bathroom fixture and bed table in the RRW and RRA, the chair handles/table and TV remote in the CA, and the food cart handle and chair handles/table in the DA. The environmental testing occurred pre- and postinstallation of the AAPT and each location tested included multiple surface samples for viable bacteria and fungus. The surface samples were taken on various surfaces in each location deemed the most commonly touched by residents and staff.

**Table 1.** Sampling Location Dimensions

Location	Length (ft)	Width (ft)	Height (ft)	Square Footage (sq. ft)
Resident room annex	17	12	8	1 858
Resident room wing	20	18	8	3 226
Dining area	33	28	8	8 242
Community area	23	17	9	3 832

The viable bacteria by air and viable fungi by air samples were completed using EMSL, a third-party laboratory specializing in biological testing (24). EMSL’s proprietary methods were used for the biological airborne testing with MICRO-SOP-132 being utilized for the bacterial testing and MICRO-SOP-202 being utilized for the fungal testing. These methods quantify and identify the 5 most concentrated species of bacteria or fungi depending on the method used. For each airborne biological test, the SOPs issued by the third-party laboratory were followed and a Viable Andersen Cascade Impactor and calibrated pump were used. The samples were taken with the pump set at 28 liters per minute for 5 minutes. To collect the samples, a soy agar plate was used for the bacterial testing and a malt extract agar plate was used for the fungal testing.

The viable bacteria by swab and viable fungi by swab samples were also completed using EMSL as the third-party laboratory (24). EMSL’s method MICRO-SOP-132 was used for the bacterial swabs and method MICRO-SOP-202 was used for the fungal swabs. These methods quantify and identify the 5 most concentrated species of bacteria or fungi depending on the method used. For each surface sample, the SOPs issued by the third-party laboratory were followed and a sterile swab was used to sample a 2-by-2-inch area on the selected sample surface for 10 seconds.

The VOC load was measured and quantified in each room utilizing the EPA TO-15 methodology (25). The third-party laboratory selected to analyze the samples was SGS Galson. This test method utilizes laboratory-prepared stainless steel canisters and regulators to pull an air sample for 4 hours. Once the air sample is returned to the laboratory, it is analyzed using gas chromatography/mass spectrometry to identify and quantify the specific VOCs present in the sample.

Paired *t*-test analyses were used to assess pre- and postinstallation airborne and surface bacterial, fungal, and VOC metrics. A *p* value of <.01 was considered statistically significant. All environmental data were analyzed and provided by 2 independent third-party industrial hygienist firms. Clinical data were collected and prospectively evaluated between the control floor and the study floor and retrospectively analyzed pre- and postinstallation. All patients and staff on both floors were included in the study, with no exclusions. Each floor had a total of 38 residents and 14 staff members during the study period. One-way analysis of variance was used to test differences in number of residents per floor, resident age, etiology, sex, and race between each study arm. Analysis of covariance was used to test differences in HAI and staff callouts. Nonparametric tests were used to assess the differences in the medians of HAIs and staff callouts between study arms. Adjusted odds ratios and 95% confidence intervals (odds ratio, 95% confidence interval) were calculated. Variables that met the statistically significant threshold of 0.20 in bivariate tests were included in the models. Unless otherwise specified, significance was set at alpha <0.01 with a 99% confidence interval. All statistical analyses were performed using SPSS 24.0 (IBM, Armonk, NY), and the study was initially powered at 90%. The control floor was used as the reference point for all data indexing and the research team was not required to normalize the data because there were no statistically significant differences found in the demographics. All residential, staff, and HAI data were provided by the LTCF and analyzed by an independent third-party epidemiologist.

## Results

The analysis of the resident demographics on the control and study floor is shown in Table 2. The demographics were analyzed to determine if there were any other factors influencing the results other than the implementation of the AAPT. The number of residents and demographics were comparable between floors and the lack of statistical significance indicates that the 2 floors were equivalent.

**Table 2.** Resident Demographics for Control and Study Floors

	Control Floor	Study Floor	*p* Value
Mean number of residents during the study	36.5	37.2	NS
Mean age (years)	88.3	89.6	NS
Etiology—% dementia	78.4	94.6	NS
% Female	78.4	73.0	NS
% Male	21.6	27.0	NS
% Hispanic	3.0	3.1	NS
% African American	2.4	—	NS
% UND	—	5.0	NS
% Caucasian	94.6	91.9	NS

*Notes*: NS = not significant; UND = undetermined race.

**Figure 2.**
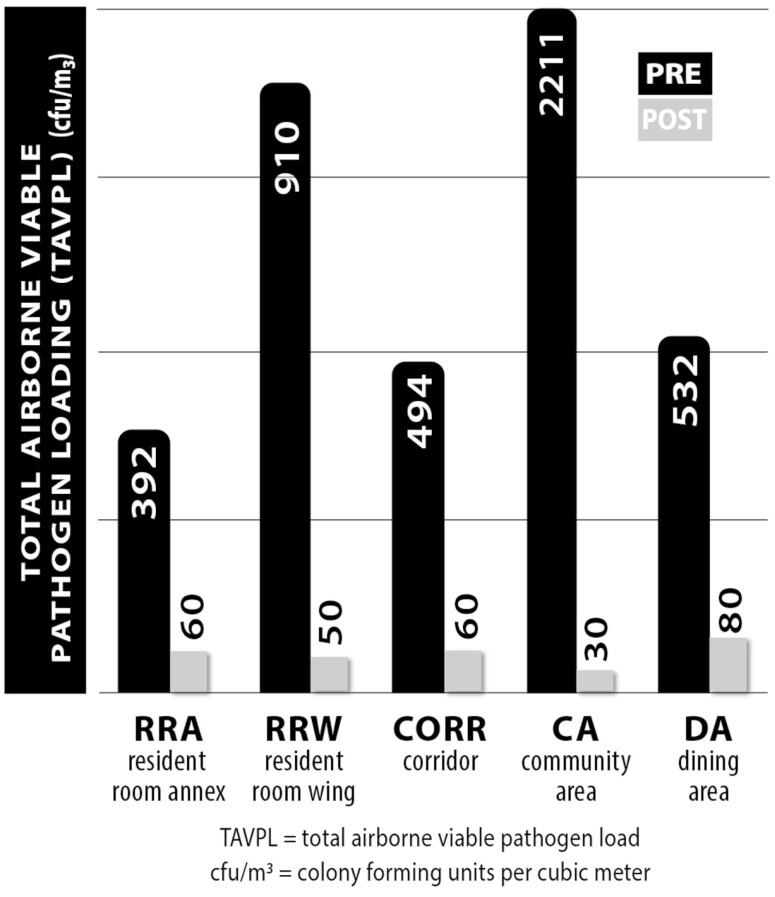
Total volatile organic compound load by location.

The overall environmental results pre- and postinstallation are presented in Table 3 and Figure 2. All data were analyzed by independent third-party laboratories after the airborne bacterial and fungal samples and VOC canister samples were taken. The total airborne viable pathogen load (TAVPL) data for the control and the study floor pre- and postinstallation is presented in Table 3. The data show that there were no statistically significant changes between the control floor pre- versus postinstallation with the exception of the RRW. This lack of statistical significance confirms that the control floor remained unchanged pre- versus postinstallation and is therefore a suitable control for the study. The data also show no statistically significant differences between the control floor and the study floor pre-AAPT installation with the exception of the CORR and CA. This lack of statistical significance also confirms that the study floor and control floor were sufficiently equivalent to preinstallation. The data show a dramatic reduction in both TAVPL and VOC levels post-AAPT installation, as compared to the preinstallation values, in every location. There was a 98.83% reduction in airborne biological and fungal pathogens across all sample locations and an 89.88% reduction in total VOC loading across the sample locations. There was also an 85.76% reduction in TAVPL between the control floor and the study floor postinstallation.

**Table 3.** Total Airborne Viable Pathogen Load (cfu/m^3^) Control Versus Study Floor and Pre- Versus Postinstallation

	Control Floor—Pre	Control Floor—Post	Study Floor—Pre	Study Floor—Post
RRA	336	322	392	60**
RRW	1 358	315*	910	50**
CORR	1 077	842	494***	60**
CA	231	212	2 211***	30**
DA	329	275	532	80**

*Notes*: CA = community area; CORR = corridor; DA = dining area; RRA = resident room annex; RRW = resident room wing.

*Statistically significant at *p* < .01 comparing control floor pre versus post.

**Statistically significant at *p* < .01 comparing study floor pre versus post.

***Statistically significant at *p* < .01 comparing control versus study floor pre-metrics.

**Figure 3.**
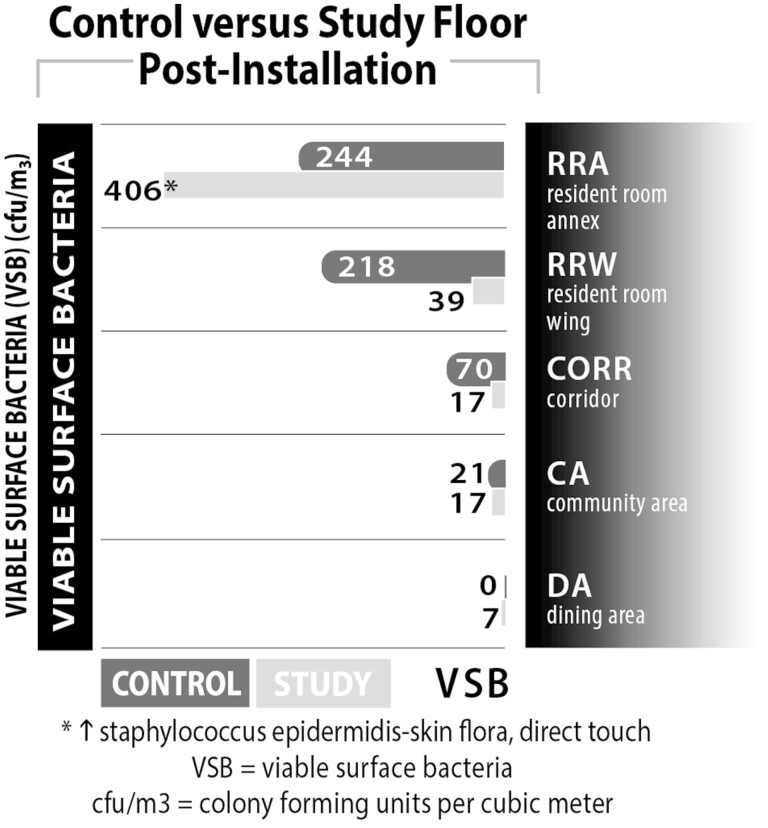
Total surface pathogen load by location.

The surface pathogen data from the commonly touched surfaces in each sampling location are presented in Figure 3. This data compare the control floor to the study floor postinstallation and sums all of the individual samples taken in each individual location. The viable surface bacteria in each location was significantly lower, *p* < .01, on the study floor than the control floor with the exception of the RRA sampling location. *Staphylococcus epidermidis* was identified in the surface samples collected in this location. This pathogen is commonly found on human skin and mucous membranes and is known to cause nosocomial infections (26). It is highly likely, based on the nature of these bacteria, that it was identified on a surface within the RRA as a result of direct skin contact by a resident or staff member and it is unlikely that it originated from the air. Because the AAPT remediates contaminants from the air before they enter a critical space, it would not be able to completely eliminate pathogen transfer between residents and staff and surfaces they touch.

The surface pathogen data were also analyzed for the study floor pre- versus postinstallation. These data are shown in Table 4. The surface bacteria in the RRW, CORR, and CA were statistically significant, *p* < .01, postinstallation as compared to preinstallation. There was a statistically significant increase in surface pathogens in the RRA postinstallation. As described previously, it is likely that this pathogen was present as a result of direct transfer from a resident or staff member.

**Table 4.** Surface Pathogens on Study Floor: Pre- Versus Postinstallation

	Study Floor—Pre	Study Floor—Post
RRA	274	406**
RRW	223	39*
CORR	74	17*
CA	35	17*
DA	10	7

*Notes*: CA = community area; CORR = corridor; DA = dining area; RRA = resident room annex; RRW = resident room wing.

*Statistically significant at *p* < .01 comparing study floor pre versus post.

**Statistically significant difference—single surface pathogen is *Staphylococcus epidermidis*.

The HAI rate in the LTCF was calculated by dividing the total number of infections by the total number of patient days. The preinstallation HAI rate was 17.3%. A prospective evaluation comparing the HAI rate for the control floor to the study floor was performed and showed a 39.6% reduction in HAIs. An additional retrospective evaluation was also performed comparing the HAI rates on the study floor preinstallation to postinstallation and this evaluation showed a 53.5% reduction in HAIs on the study floor. The pre- versus postinstallation and study period number of HAIs are presented in Table 5. To confirm that there were no outside factors affecting the HAI rates during the study period, the pre- and postinstallation HAI rates on the control floor were examined. This analysis yielded no statistically significant difference in the control floor HAI rates pre- versus postinstallation. Though the research team did not include staff callouts as a metric in the study, the data analysis revealed a 47% statistically significant, *p* < .01, reduction in staff callouts pre- versus postinstallation.

**Table 5.** Total Health Care-Associated Infections (HAIs) Reported

	Control Floor	Study Floor
Preinstallation HAIs	41	44
Postinstallation HAIs	43	20
Study period HAIs	53	32

*Note*: Study period = October 2019 to January 2021.

## Discussion

Indoor air quality is a critical variable that must be controlled in order to create a safe and healthy environment for health care facilities (27). The link between building ventilation systems and the transmission or spread of infectious diseases has been well-documented (28). It is therefore necessary to provide high-quality and reliable air filtration to protect LTCFs. The current study focuses on an AAPT integrated into the ductwork of an LTCF and the relationship between environmental purity and clinical outcomes.

There are no known studies that evaluate the effectiveness of similar air purification technologies within the LTCF environment. UVGI applications within LTCFs typically focus on the use of UVGI to disinfect surfaces and high-touch locations in conjunction with regular cleaning (29). These applications require the spaces to be vacant during disinfection and do not provide protection for pathogens that are generated between cleaning events. The use of filter media to remove VOCs has been widely accepted in other applications, but has not been studied within the LTCF environment. The AAPT in the current study is novel and unique in its application to both pathogen and VOC remediation.

Infection control initiatives within the LTCF environment, typically focus on hand washing and surface cleaning. Multiple LTCF studies have confirmed the effectiveness of hand washing on eliminating pathogens from hands and reducing infection rates (30–34). Though this infection control method is effective, it is well-documented that hand hygiene compliance within these facilities is low and averages 17% (35). Similarly, there are many studies that demonstrate the effectiveness of surface disinfection in the reduction of pathogens and possibly the reduction of HAIs (34,36–39). Though surface cleaning is effective, there is also great variation in cleaning practice between facilities and the individuals performing the cleaning (40). Hand hygiene and surface disinfection are important facets of any infection control program, but they rely on individual compliance and technique and can therefore vary in their effectiveness. In contrast, the AAPT in the present study is part of the LTCFs HVAC infrastructure and operates independent of human intervention and its effectiveness is therefore not reliant on compliance or technique from LTCF staff. This study demonstrates that in an LTCF with similar floor layouts, staffing, resident populations, and cleaning protocols, the use of the AAPT resulted in decreased viable surface and airborne pathogen loading, VOC levels, HAI rates, and staff callouts.

The environmental testing performed preinstallation and on the control floor indicate the presence of airborne and surface pathogens present within the LTCF. The presence of these pathogens denotes an opportunity to improve resident wellness and safety because these microbials have the potential to cause illness or infection to residents, staff, or visitors (41). Infectious airborne pathogens can also settle onto surfaces, posing a risk of infection to individuals who come in contact with contaminated surfaces (42). The installation of the AAPT dramatically reduced airborne pathogens on the study floor pre- and postinstallation. Not only was there a reduction in airborne pathogens, but there was also a concomitant reduction in surface pathogens measured on commonly touched surfaces across the rooms studied. The reduction in surface pathogens was a direct result of the reduced pathogen load in the air and provided valuable insight into the understanding of how airflow interacts with aerosolized pathogens.

The reduction in airborne and surface pathogens and total VOC load translated to a statistically significant 39.6% reduction in HAIs between the study floor and the control floor. Older adults have underlying conditions and compromised immune systems that increase their likelihood of infection, making them especially susceptible to contracting illness or infection (5,10). By reducing HAIs, an LTCF can have an immediate and direct impact on resident quality of life and wellness. In addition to benefits to the residents, the LTCF is also likely to experience improvements in their preferred provider ratings, increased referrals, improved payer status, and improvements in reimbursements (43).

The statistically significant reduction in both HAIs has clear economic impacts because there is a direct link between HAI rates and reimbursements from the Centers for Medicare and Medicaid Services (CMS). CMS pays the LTCFs a fixed amount per day per resident and withholds 2% of their total payment. The LTCFs have the opportunity to earn back all or a portion of the withheld payment based on their health care performance. CMS ranks health care performance according to the LTCF’s hospital readmission rates. CMS evaluates facilities’ year-over-year improvement and also takes into account facilities that rank in the top half of all facilities in the United States. The 39.6% reduction in HAIs following the installation of the AAPT will not only yield a year-over-year improvement for the facility but it will also increase the facility’s overall ranking, thus maximizing their reimbursement. Minimizing HAIs has a further positive economic impact because when an LTCF resident is taken to the hospital for an HAI, the LTCF loses their daily CMS payment for the duration of their hospital stay.

The 47% reduction in staff callouts demonstrated by the AAPT has positive clinical impacts for the LTCF. When a staff member calls out, the LTCF must replace this individual by extending the shift of another employee, bringing in an employee who was scheduled to be off, or hiring an agency nurse. Agency nurses not only cost more than staff nurses but also disrupt the continuity of clinical care causing care failures. The agency nurses are not familiar with the residents and the use of these nurses has been linked to increased patient falls, medication errors, meal errors, and a negative impact on the mental well-being of the residents. Avoiding staff callouts can have a positive impact on resident wellness and help to minimize care failures.

The findings in the LTCF are not unique and improved air quality has been linked to benefits in other health care settings. Other studies utilizing a similar AAPT from the same company have been performed in a separate health care facility, namely an acute care hospital with similar results. These studies have noted statistically significant reductions in airborne and surface pathogens and concomitant improvements in clinical and economic metrics (44,45).

This study has several important limitations. First, the clinical data that were analyzed pre- and postinstallation were analyzed retrospectively and contained biases inherent to retrospective study designs. Secondly, the present study was not designed to analyze specific clinical outcomes such as the number of hospitalizations for specific diagnoses. The study reported total HAIs but the data collected by the LTCF did not provide granularity to the underlying cause of the HAI reported.

It is particularly important to protect older adults, who have more susceptible immune systems and reside in LTCFs, from airborne infectious agents. The AAPT offers an effective and safe solution to remediating all airborne contaminants including VOCs, viable particulates, and nonviable particulates in order to create a safe indoor environment for residents, staff, and visitors. The AAPT has been associated with statistically significant reductions in infectious airborne pathogens and the associated improvements in clinical outcomes such as HAI rates. The study findings support the hypothesis that the comprehensive remediation of all airborne contaminants would reduce airborne and surface pathogen loads, VOC loads and have a positive impact on HAI rates. The use of the AAPT could be an important addition to current infection control programs to improve overall cleanliness and reduce infections within LTCFs. Further research into indoor air quality within the LTCF environment is warranted. Different types of LTCFs could be studied to understand the impact of the AAPT across LTCF environments.
